# Identification and Validation of a Malignant Cell Subset Marker-Based Polygenic Risk Score in Stomach Adenocarcinoma Through Integrated Analysis of Bulk and Single-Cell RNA Sequencing Data

**DOI:** 10.3389/fcell.2021.720649

**Published:** 2021-10-18

**Authors:** Qiyuan Zou, Yufeng Lv, Zuhuan Gan, Shulan Liao, Zhonghui Liang

**Affiliations:** ^1^Department of Gastroenterology, Foresea Life Insurance Guangxi Hospital, Nanning, China; ^2^Center of Oncology, Foresea Life Insurance Guangxi Hospital, Nanning, China

**Keywords:** stomach adenocarcinoma (STAD), single-cell RNA sequencing (scRNA-seq), intratumoral heterogeneity (ITH), polygenic risk score (PRS), pseudotime analysis

## Abstract

**Objectives:** The aim of the present study was to construct a polygenic risk score (PRS) for poor survival among patients with stomach adenocarcinoma (STAD) based on expression of malignant cell markers.

**Methods:** Integrated analyses of bulk and single-cell RNA sequencing (scRNA-seq) of STAD and normal stomach tissues were conducted to identify malignant and non-malignant markers. Analyses of the scRNA-seq profile from early STAD were used to explore intratumoral heterogeneity (ITH) of the malignant cell subpopulations. Dimension reduction, cell clustering, pseudotime, and gene set enrichment analyses were performed. The marker genes of each malignant tissue and cell clusters were screened to create a PRS using Cox regression analyses. Combined with the PRS and routine clinicopathological characteristics, a nomogram tool was generated to predict prognosis of patients with STAD. The prognostic power of the PRS was validated in two independent external datasets.

**Results:** The malignant and non-malignant cells were identified according to 50 malignant and non-malignant cell markers. The malignant cells were divided into nine clusters with different marker genes and biological characteristics. Pseudotime analysis showed the potential differentiation trajectory of these nine malignant cell clusters and identified genes that affect cell differentiation. Ten malignant cell markers were selected to generate a PRS: RGS1, AADAC, NPC2, COL10A1, PRKCSH, RAMP1, PRR15L, TUBA1A, CXCR6, and UPP1. The PRS was associated with both overall and progression-free survival (PFS) and proved to be a prognostic factor independent of routine clinicopathological characteristics. PRS could successfully divide patients with STAD in three datasets into high- or low-risk groups. In addition, we combined PRS and the tumor clinicopathological characteristics into a nomogram tool to help predict the survival of patients with STAD.

**Conclusion:** We revealed limited but significant intratumoral heterogeneity in STAD and proposed a malignant cell subset marker-based PRS through integrated analysis of bulk sequencing and scRNA-seq data.

## Introduction

Stomach adenocarcinoma (STAD) is the most frequent histological type of stomach cancer and the fifth most common type of cancer. It is the third most lethal cancer worldwide (Bray et al., 2018; Rawla and Barsouk, 2019). Poor prognosis of STAD patients results from multiple factors, such as late clinical presentation, genetic heterogeneity, and effective drug resistance. Currently, some classification systems based on histological or genetic characteristics of STAD aim to identify high-risk patients and provide personalized treatment. The Lauren classification system divides STAD into the diffuse (poorly differentiated) subtype, the intestinal (well differentiated) subtype, and the mixed type (Lauren, 1965). The Cancer Genome Atlas (TCGA) Research Network reported the following four subtypes of STAD based on genomic characteristics: EBV-positive (9%), microsatellite instable (MSI) (22%), genomically stable (20%), and chromosomally instable (50%) (Cancer Genome Atlas Research Network, 2014). Similar results were also seen in studies from Singapore (Lei et al., 2013) and the Asian Cancer Research Group (Cristescu et al., 2015). These classification systems may lead to the development of specific therapies. For example, patients with EBV-positive or MSI–high STAD may not benefit from adjuvant chemotherapy (Ramos et al., 2020), but they may benefit from immune checkpoint inhibitors (Derks et al., 2016; Muro et al., 2016). These classification systems are based on data derived from bulk tissues, so they cannot capture intratumoral heterogeneity (ITH). Increasing evidence shows that tumors harbor various genetic subpopulations that differ in their response to drug therapies (Saunders et al., 2012). Indeed, complete responses to drug therapies are rare in solid tumors. Partial responses and secondary resistance indicate that some but not all subpopulations in a given tumor are sensitive to therapy.

Recently, single-cell RNA sequencing (scRNA-seq) provides methods to characterize the transcriptional state of thousands of individual cells and perform an unbiased analysis of cellular characteristics (Wen and Tang, 2016). It has been widely used to dissect ITH in various cancers (Levitin et al., 2018; Gonzalez-Silva et al., 2020) including STAD (Zhang et al., 2021). However, the ITH of early-stage STAD is poorly understood. Early STAD involves invasion of the mucosa and submucosa (T1), irrespective of lymph node metastases (any N) (Gotoda, 2006). In our present study, we reanalyzed the scRNA-seq data from a sample with early STAD in order to reveal the ITH. Furthermore, we combined this information with that derived from bulk STAD tissue gene expression profiles to create a marker-based polygenic risk score (PRS) that can help identify STAD patients at high risk of poor survival.

## Materials and Methods

### Expression Datasets

The following public expression datasets were used in this study:

#### Expression Datasets From Bulk Tissue

Datasets GSE66229, GSE113255, GSE84437, and GSE26942 were obtained from Gene Expression Omnibus.^1^ All datasets were generated using microarrays. The data were downloaded as originally normalized by the authors and further processed as follows: if a gene was detected by several probes, the expression of that gene was defined as the average value of the gene calculated across all the probes. GSE66229 comprises expression data from 300 STAD and 100 normal mucosa tissues (Oh et al., 2018), GSE113255 contains expression data from 130 STAD and 10 mucosa tissues (Kim et al., 2020), GSE84437 contains gene expression profiles and overall survival (OS) information of 433 STAD tissues (Yoon et al., 2020), and GSE26942 contains gene expression profiles and OS information of 202 STADs (Oh et al., 2018). We first removed 3 gastrointestinal stromal tumors and 12 surrounding normal gastric tissues from GSE26942 before analysis.

The TCGA-STAD dataset was downloaded from TCGA repository.^2^ It consists of bulk RNA-seq data (displayed as raw read counts) from 375 STAD and 32 normal tissues together with clinical information. Raw read counts were normalized using the voom function in the limma package in R.

#### Expression Dataset From Single Cells

The GSE134520 complete dataset was available in Gene Expression Omnibus (see text footnote 1) and comprises scRNA-seq data from 13 stomach mucosa biopsies from nine patients with non-atrophic gastritis, chronic atrophic gastritis, or intestinal metaplasia and one patient with early-stage STAD (Zhang et al., 2019a). Only the data from the early-stage STAD sample (GSM3954958) were downloaded for the present study. The scRNA-seq data had been generated using the 10X Genomics platform and were further processed in the present study as indicated below.

**FIGURE 1:**
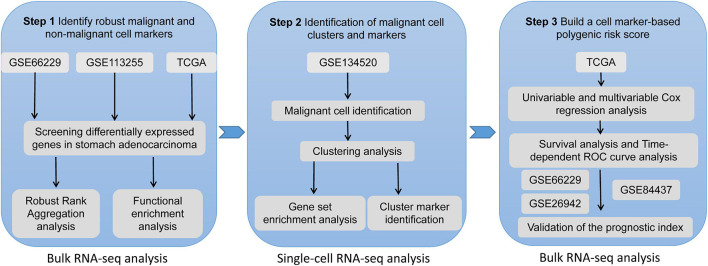
The workflow of the present study.

The datasets used in our study are publicly available, so no further ethical approval was necessary for the present study. A detailed workflow of the use of the datasets in our study can be seen in Figure 1.

##### Identification of robust malignant and non-malignant cell markers

For GSE66229 and GSE113255, which are based on microarray platforms, differential expression analysis was performed using the limma package (Ritchie et al., 2015). Differentially expressed genes (DEGs) from the TCGA-STAD RNA-seq dataset were identified using the DESeq2 package (Love et al., 2014) based on a criterion of *p* < 0.05 after adjustment by the false discovery rate (FDR). The DEGs in the individual dataset were then integrated and ranked with the RobustRankAggreg package (Kolde et al., 2012) to obtain robust malignant and non-malignant cell markers. Briefly, the DEGs were ranked first based on log_2_(fold change) in individual datasets, and then the three ranked lists were subjected to robust rank aggregation analysis. Based on ranking by *p* value, the top 50 significantly upregulated genes were considered as malignant cell markers, and the top 50 significantly downregulated genes were considered as non-malignant cell markers (Zhang et al., 2021).

##### Kyoto encyclopedia of genes and genomes

To uncover the potential biological functions of malignant and non-malignant cell markers, Kyoto Encyclopedia of Genes and Genomes (KEGG) pathway enrichment analysis was performed using the clusterProfiler package (Yu et al., 2012).

##### Single-cell RNA-sequencing data

###### Preprocessing

The raw gene expression matrices from early STAD tissues (GSM3954958 samples from the GSE134520 dataset) were imported and processed using the Seurat R package (version 3.2.0)^3^ (Butler et al., 2018; Stuart et al., 2019) and preprocessed as follows. First, low-quality cells were removed based on one of these three criteria: (1) number of expressed genes lower than 200; (2) a number of expressed genes larger than 6,000; or (3) 50% or more of unique molecular identifiers (UMIs) mapped to mitochondrial genes (Supplementary Figure 1A). Usually, cells are considered as low-quality when more than 15–25% of UMIs map to mitochondrial genes. Here we used the 50% mapping cutoff because stomach tissue is metabolically active and the gastric epithelium is expected to have high mitochondrial content. The gene expression profiles of the cells that passed this quality cutoff (3,771 cells) were then normalized using *normalization.method* = “*LogNormalize.*”

Next, each of the 3,771 cells was annotated as malignant or non-malignant/unknown using the SCINA R package (Zhang et al., 2019c), and the expression of malignant and non-malignant cell markers was determined as described above.

##### Dimension reduction and cell clustering analysis of malignant cells

Cells identified as malignant were subjected to subsequent analysis. The top 2,000 genes with the largest variance were selected as highly variable genes (HVGs) using Seurat “FindVariableGenes” function and used for further analyses (Supplementary Figure 1B). The expression profiles were centered and scaled values using “ScaleData” function before performing dimension reduction and clustering analysis. The “RunPCA” function in the Seurat package was used to carry out principal component analysis (PCA) on the scRNA-seq expression matrix of HVGs. The top 20 principal components (PCs), which explained most of the variance, were subjected to further analysis (Supplementary Figure 1C). Then, the “FindClusters” function in the Seurat package was utilized to perform cell clustering analysis, and the parameter resolution was set as 0.5. Furthermore, uniform manifold approximation and projection (UMAP) dimensionality reduction was conducted and visualized using the RunUMAP function in the Seurat package. Cell cluster marker genes were identified using the “FindMarkers” function with the following parameters: only.pos = F, min.pct = 0.25, logfc.threshold = 0.5, and test.use = “roc.”

##### Pseudotime analysis

A malignant tumor has highly heterogeneous cell populations. Investigation of the differentiation trajectories and corresponding genes in the various cell populations may clarify the molecular mechanisms of cancer development. Pseudotime and cell trajectory analyses were carried out using the Monocle R package (Qiu et al., 2017) and default parameters.

##### Gene set enrichment analysis

Gene set enrichment analysis (GSEA; Subramanian et al., 2005) was performed to characterize biologically the malignant cell clusters using the SingleSeqGset package.^4^ SingleSeqGset is a package for GSEA for scRNA-seq data. It uses variance-inflated Wilcoxon rank sum testing to determine enrichment of gene sets of interest across clusters. *p* < 0.05 after adjustment by the FDR was considered significant.

##### Univariable and multivariable cox regression and polygenic risk score

The expression profiles of malignant markers and the marker genes of the malignant cell clusters were first used to perform univariable Cox regression analysis in the TCGA-STAD normalized data. Significant genes (*p* < 0.05) were then subjected to multivariate Cox regression. Next, regression analysis was run to create a PRS using the following formula:


PRS=Expr×g⁢e⁢n⁢e1β+g⁢e⁢n⁢e1Expr×g⁢e⁢n⁢e2β+g⁢e⁢n⁢e2

Expr×g⁢e⁢n⁢e3β+g⁢e⁢n⁢e3…

where Expr represents the expression value of the genes in the multivariate Cox regression analysis, and β is the corresponding estimated regression coefficient.

##### Analyses of time-dependent receiver operating characteristic curves and survival

Time-dependent receiver operating characteristic curve (tROC) curve analysis was performed using the tROC package in R (Blanche et al., 2013). In brief, TCGA samples for which clinical annotation was available were divided into low- or high-risk groups, based on the median PRS. The OS and progression-free survival (PFS) between the low- and high-risk groups were compared using the log-rank method.

##### Nomogram model

The PRS was combined with routine clinicopathological features (available for the dataset) to create a nomogram model in order to better predict the prognosis of STAD patients. The nomogram was created using the rms package^5^ in R.

##### Validation of the polygenic risk score

Four datasets (GSE84437, GSE66229, and GSE26942) were used to validate the prognostic value of the PRS. If the PRS was significantly associated with OS, but the median PRS in the dataset failed to divide patients into high- or low-risk groups, the optimal cutoff was identified using the survminer package.^6^

## Results

### Robust Stomach Adenocarcinoma and Non-malignant Cell Markers

**FIGURE 2:**
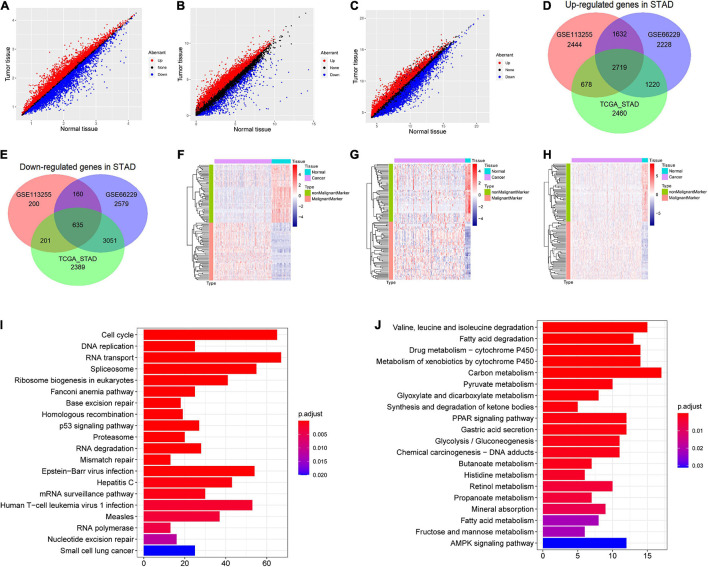
Screening differentially expressed genes (DEGs) in stomach adenocarcinoma (STAD) and malignant and non-malignant cell markers. **(A)** DEGs in GSE66229; **(B)** DEGs in GSE113255; **(C)** DEGs in The Cancer Genome Atlas (TCGA)-STAD dataset. **(D)** Venn plot of overlap up-regulated genes in the three dataset. **(E)** Venn plot of overlap down-regulated genes in the three dataset. **(F)** Expression heat map of malignant and non-malignant cell markers in GSE66229. **(G)** Expression heat map of malignant and non-malignant cell markers in GSE113255. **(H)** Expression heat map of malignant and non-malignant cell markers in TCGA-STAD dataset. **(I)** The top 20 significant Kyoto Encyclopedia of Genes and Genomes (KEGG) pathways (ranked by *p* value) involving the overlapping up-regulated genes. **(J)** The top 20 significantly KEGG pathways (ranked by *p* value) involving the overlapping down-regulated genes.

In GSE66229 dataset, a total of 14,224 DEGs were identified, including 7,799 up-regulated and 6,425 down-regulated in STAD (Figure 2A). In GSE113255, a total of 8,669 DEGs were identified, including 7,473 up-regulated and 1,196 down-regulated in STAD (Figure 2B). In TCGA-STAD, a total of 13,353 DEGs were identified, including 7,077 up-regulated and 6,276 down-regulated in STAD (Figure 2C). Notably, the up-regulated (Figure 2D) and down-regulated genes (Figure 2E) varied substantially across the three datasets. Thus, identifying robust markers was necessary. After robust rank aggregation analysis, we selected 50 robust malignant markers and 50 robust non-malignant markers (Supplementary Table 1). In expression heat maps (Figure 2F for GSE66229, Figure 2G for GSE113255, and Figure 2H for TCGA-STAD), these 100 genes showed clearly different expression patterns between STAD and normal stomach tissues across the three datasets. In addition, the overlapping up- and down-regulated genes involved different KEGG pathways. Figure 2I shows the top 20 KEGG pathways (ranked by *p* value) involving overlapping up-regulated genes, which included the cell cycle, p53 signaling pathway, and Epstein–Barr virus infection. Figure 2J shows the top 20 KEGG pathways involving the overlapping down-regulated genes, which included the peroxisome proliferator-activated receptor (PPAR) signaling pathway, gastric acid secretion, and AMPK signaling pathway.

### Intratumoral Heterogeneity in Early-Stage Stomach Adenocarcinoma Tumors

**FIGURE 3:**
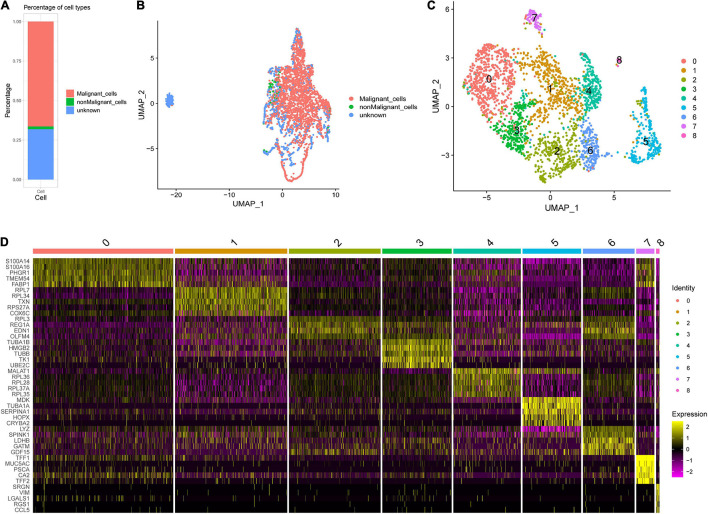
Single-cell RNA sequencing (scRNA-seq) analysis in an early STAD. **(A)** Percentages of cell types identified by SCINA package. **(B)** Scatterplot for uniform manifold approximation and projection (UMAP) using the malignant and non-malignant marker genes. **(C)** The UMAP plot of malignant cells, showing cell clusters. **(D)** The relative expression heat map for malignant cell cluster markers. Only the top five are shown.

**FIGURE 4:**
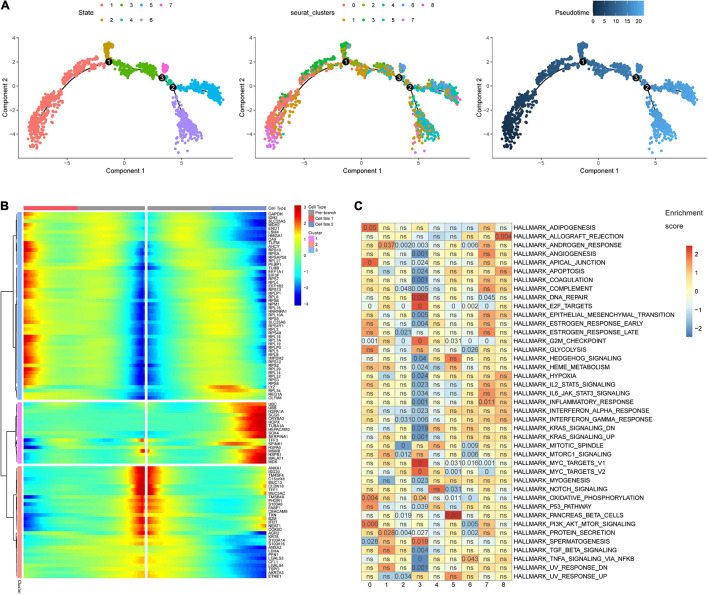
Pseudotime analysis and gene set enrichment analysis (GSEA). **(A)** The malignant cell differentiation trajectory plot, **(B)** The heat map for the expression patterns of the top 100 significant genes (ranked by *q* value) in branched expression analysis modeling (BEAM). **(C)** The eight malignant cell clusters were enriched in various hallmark gene sets. The number in the grids refers to the *p* value adjusted by the false discovery rate (FDR).

The 3,771 cells remaining after quality control were identified by the SCINA package as 2,506 malignant cells, 63 non-malignant cells, and 1,202 unknown type cells based on the malignant and non-malignant cell markers (Figure 3A). These three types of cells were not well distinguished by PCA based on the expression patterns of the 100 marker genes (Figure 3B). The malignant cells were further identified as nine cell clusters (Figure 3C). The cell cluster markers were screened (Supplementary Table 2), and the top five positive markers (ranked by logFC) were used to draw an expression heat map (Figure 3D). Notably, most cluster markers were not included among the overlapping up- or down-regulated genes in STAD (Supplementary Figure 2A), and few malignant and non-malignant cell markers were included among the malignant cell cluster markers (Supplementary Figure 2B). The pseudotime analysis was performed using the cell cluster markers. The analysis suggested that the potential cell differentiation trajectories of the malignant cells comprised seven states (Figure 4A). In addition, we also performed branched expression analysis modeling (BEAM) analysis to identify the cell cluster marker genes that change as cells pass from the early developmental stage to the top left of the tree through the branch using the “BEAM” function. The significant DEGs between the branches (*q* < 0.05) were included in Supplementary Table 3. The expression patterns of the top 100 significant genes (ranked by *q* value) are shown as a heat map in Figure 4B.

The GSEA indicated that the eight cell clusters were enriched in various hallmark gene sets (Figure 4C). Cell cluster 0 was significantly enriched in PI3K/AKT/MTOR signaling and oxidative phosphorylation. Cell cluster 1 seems to harbor stronger protein secretion ability and was significantly enriched in the protein secretion hallmark gene set. The down-regulated genes of cell cluster 2 were significantly enriched in hallmark gene sets of G2M checkpoint, E2F targets, and protein secretion. The hallmark gene sets MYC targets, DNA repair, and E2F targets were significantly enriched in cell cluster 3. The hallmark gene sets of pancreatic beta cells, tumor necrosis factor α signaling *via* nuclear factor κβ, inflammatory response, and allograft rejection were significantly enriched, respectively, in cell clusters 5, 6, 7, and 8. The results indicate that ITH emerges in the early stage of STAD. Biological heterogeneity in STAD subpopulations may be the basis of drug resistance.

### A Cell Marker–Based Polygenic Risk Score for Predicting Prognosis in Stomach Adenocarcinoma

**FIGURE 5:**
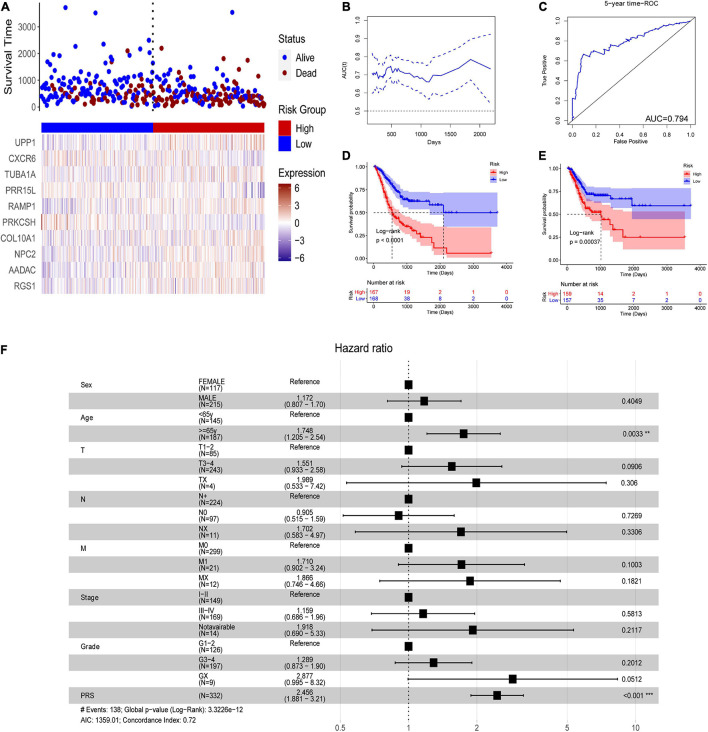
The polygenic risk score (PRS) in the TCGA-STAD dataset. **(A)** Patients with STAD were divided into high- or low-risk groups according to the median PRS. **(B)** The areas under time-dependent receiver operating characteristic (ROC) curves. **(C)** 4-year ROC curve of the PRS. **(D)** overall survival (OS) analysis using Kaplan–Meier curves with the log-rank test. **(E)** progression-free survival (PFS) analysis using Kaplan–Meier curves with the log-rank test. **(F)** Multivariable Cox analysis of the PRS and routine clinicopathological characteristics. **P < 0.01; ***P < 0.001.

**FIGURE 6:**
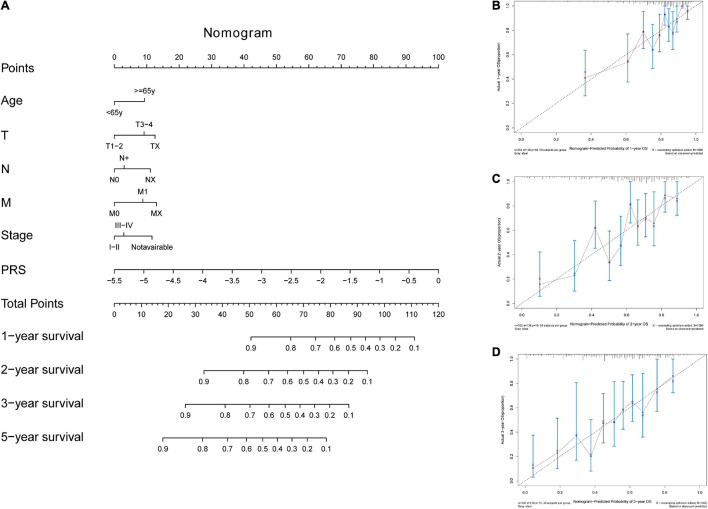
The nomogram tool for predicting survival rate in the TCGA-STAD dataset. **(A)** The nomogram tool. **(B)** Calibration curves for 1-year, **(C)** 2-year, and **(D)** 3-year survival rates with the nomogram tool.

**FIGURE 7:**
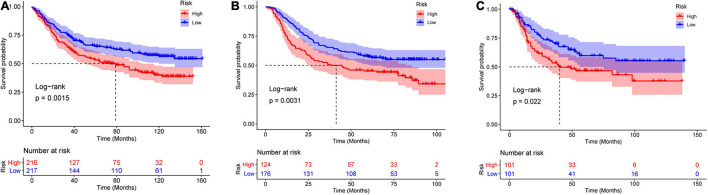
Validation of the PRS against the **(A)** GSE84437, **(B)** GSE66229, and **(C)** GSE26942 datasets.

In the TCGA-STAD dataset, the malignant marker genes and the eight malignant cell cluster marker genes were used to perform univariable Cox analysis, and 38 genes were significantly associated with OS. Ten of them (RGS1, AADAC, NPC2, COL10A1, PRKCSH, RAMP1, PRR15L, TUBA1A, CXCR6, and UPP1) were retained to construct the PRS through univariable and multivariable Cox proportional hazards regression and stepwise regression (Supplementary Table 4). The PRS showed significant association with OS [hazard ratio (HR) = 2.1356, 95% CI = 1.6466–2.7697, *p* < 0.0001, Figure 5A]. Continuous tROC curve analysis (Figure 5B) showed that the PRS may perform well at predicting 5-year OS, with an area under the ROC curve (AUC) = 0.794 (Figure 5C). The PRS was also associated with PFS (HR = 2.7183, 95% CI = 2.1065–3.5078, *p* < 0.0001). The patients with STAD were divided into high- or low-risk groups. The patients in the high-risk group had shorter OS (Figure 5D) and PFS (Figure 5E) than those in the low-risk group. Furthermore, the PRS was an independent prognostic factor compared with routine clinicopathological factors (Figure 5F). In addition, we combined the routine clinicopathological factors that were associated with OS to construct a nomogram model for predicting OS rate (Figure 6A), which showed a concordance index = 0.7151 (95% CI = 0.6714–0.7589). The calibration curves for OS at 1, 2, and 3 years demonstrated good agreement between prediction and observation (Figures 6B–D). The prognostic value of the PRS was validated against the data in GSE84437 (HR = 1.566, 95% CI = 1.2205–2.0102, *p* = 0.0004), GSE66229 (HR = 3.4176, 95% CI = 1.127–10.362, *p* = 0.0299), and GSE26942 (HR = 1.434, 95% CI = 1.0211–2.0156, *p* = 0.0375). The patients in the high-risk group had shorter OS than those in the low-risk group in the datasets GSE84437 (Figure 7A), GSE6229 (Figure 7B), and GSE26942 (Figure 7C).

## Discussion

Intratumoral heterogeneity includes a spatial component (heterogeneity in different tumor areas) and temporal component (heterogeneity during progression from early to advanced disease) (Gullo et al., 2018). It is a major obstacle to the success of molecular treatments (Alsina et al., 2017). In current clinical practice, despite ITH, patients with STAD are treated according to pathological staging and expression of certain cancer markers such as Hrb-b2 receptor tyrosine kinase 2 (HER2; Bang et al., 2010). Moreover, therapeutic intervention may promote tumor progression by providing selective pressure that promotes the expansion of resistant subpopulations (Kreso et al., 2013; Burrell and Swanton, 2014). ITH is the problem that must be overcome in the treatment of acquired drug-resistant tumors. In recent years, with the development of scRNA-seq technology, ITH has gradually been revealed. In the present study, we identified nine clusters of malignant cells in early STAD. The biological characteristics varied significantly among the malignant cell clusters, which implies that the cell subsets may respond differently to therapies. For example, the malignant cell cluster 0 was enriched in the PI3K/AKT/mTOR pathway and so may respond to treatments targeting this pathway. Some PI3K inhibitors are being evaluated in clinical trials (Yang et al., 2019).

At the tissue level, functional enrichment analysis of overlapping up- and down-regulated genes revealed the pathways in which these genes are involved, which included the cell cycle (Kastan and Bartek, 2004), p53 signaling pathway (Joerger and Fersht, 2016), and DNA mismatch repair (Baretti and Le, 2018). These results also reveal some pathways that may provide diagnostic biomarkers, such as Epstein–Barr virus infection, PPAR signaling pathway, and DNA replication. At the cell level, the GSEA of malignant clusters revealed some biological characteristics of the individual cell clusters. We found that the iconic cancer-related pathways, such as p53 signaling and PI3K/AKT/mTOR pathways, are not significantly enriched in all malignant clusters. These results highlight how ITH poses a challenge to optimizing multidrug combination regimens or sequential treatments.

The expression level of any single gene varies between cells, partially due to the random and noisy nature of expression regulation. Thus, it is essential to identify candidate genes that affect cell differentiation, prognosis, and treatment efficacy. HER2 and kinase insert domain receptor (KDR also known as VEGFR2) are validated therapeutic targets in STAD (Bang et al., 2010; Fuchs et al., 2014). Given ITH, more therapeutic targets are urgently needed. Several gene-based signatures have been reported in previous studies (Zhu et al., 2016; Hou et al., 2017; Zhang et al., 2019b), but they have been based on bulk RNA profiling, which averages the expression profiles of the constituent cells and therefore ignores ITH. Whether the genes included in these signatures are expressed in malignant or non-malignant cells (such as tumor-associated fibroblasts and tumor-infiltrating lymphocytes) is unknown. In the present study, the marker genes for each malignant cell cluster were identified, and some of them were also found to determine cell differentiation according to BEAM analysis. A malignant marker and malignant cell marker–based PRS was created to predict prognosis for STAD.

Ten genes were included in our PRS: RGS1, AADAC, NPC2, COL10A1, PRKCSH, RAMP1, PRR15L, TUBA1A, CXCR6, and UPP1. AADAC is a negative marker for malignant cell cluster 5 and associated with poor prognosis in STAD. Few previous studies have focused on the role of NPC2 in STAD; in the present study, NPC2 was identified as a negative marker of cell cluster 4 and was found to be up-regulated in STAD. COL10A1 may promote invasion and metastasis in STAD *via* epithelial-to-mesenchymal transition (Li et al., 2018), and here we found it to be a malignant cell marker, but not a cell cluster marker. PRKCSH containing the GAG trinucleotide repeat has been reported as a mutational target in high-MSI STAD (Palmirotta et al., 2011). RAMP1 has been found to be a cancer-promoting gene in many studies (Logan et al., 2013; Mishima et al., 2017; Dallmayer et al., 2019). RGS1 is the marker gene of malignant cell cluster 8 and has been associated with poor prognosis in the present work and in a previous study (Li et al., 2021). However, another study failed to detect such an association (Zhu et al., 2021). The inconsistency in previous studies may be attributed to the high ITH of STAD. Here we propose a PRS based on markers of malignant cell subsets. The PRS is an independent prognostic factor compared with routine clinicopathological characteristics, and it can divide patients with STAD into high- or low-risk groups. We validated the PRS in three external datasets.

Although the present study may provide new insight into STAD through integrated analysis of bulk and scRNA-seq data, it has several limitations. First, the scRNA-seq profile was from early STAD; thus, the subpopulations of malignant cells that can be identified may be limited. Second, the PRS was developed based on retrospective analysis and should be validated in prospective trials before its use in clinical practice. Third, the present study lacked molecular experiments to further explore the specific mechanism of the malignant cell markers. It is unknown whether the observed expression changes in these markers are a cause or effect of STAD cell phenotypes and patient prognosis.

Despite these limitations, our analyses reveal limited but significant ITH in early STAD. Based on integrated analysis of bulk and single-cell expression data, we propose a malignant cell subset marker-based PRS that can identify STAD patients at high risk of poor survival. The PRS, in combination with routine clinicopathological evaluation of tumors, may help clinicians provide more personalized treatment.

## Data Availability Statement

Publicly available datasets were analyzed in this study. This data can be found here: https://www.ncbi.nlm.nih.gov/geo/; https://portal.gdc.cancer.gov/.

## Author Contributions

YL and ZL designed the study. QZ and YL collected the data, performed the analysis, and wrote the manuscript. ZG and SL participated in the analysis and prepared the manuscript draft. All authors approved the final manuscript.

## Conflict of Interest

The authors declare that the research was conducted in the absence of any commercial or financial relationships that could be construed as a potential conflict of interest.

## Publisher’s Note

All claims expressed in this article are solely those of the authors and do not necessarily represent those of their affiliated organizations, or those of the publisher, the editors and the reviewers. Any product that may be evaluated in this article, or claim that may be made by its manufacturer, is not guaranteed or endorsed by the publisher.

## Footnotes

1
https://www.ncbi.nlm.nih.gov/geo/

2
https://www.cancer.gov/tcga

3
http://satijalab.org/seurat/

4
https://arc85.github.io/singleseqgset/

5
https://CRAN.R-project.org/package=rms

6
https://CRAN.R-project.org/package=survminer

## Supplementary Material

Supplementary Figure 1 **(A)** Quality control in the single-cell RNA sequencing analysis. **(B)** The HVGs and the top 20 HVGs were labeled. **(C)** The standard deviation explained by PC. **(D)** The top (ranked by log fold change) marker genes for each malignant cell clusters.

Supplementary Figure 2 **(A)** Most cluster markers were not included among the overlapping up- or down-regulated genes in STAD. **(B)** Few malignant and non-malignant cell markers were included among the malignant cell cluster markers.
