# Supplementary figures and images for: Identification and Validation of a Malignant Cell Subset Marker-Based Polygenic Risk Score in Stomach Adenocarcinoma Through Integrated Analysis of Bulk and Single-Cell RNA Sequencing Data
Source: Front Cell Dev Biol. 2021 Oct 18;9:720649. doi: 10.3389/fcell.2021.720649 (PMC8558465; doi:10.3389/fcell.2021.720649)

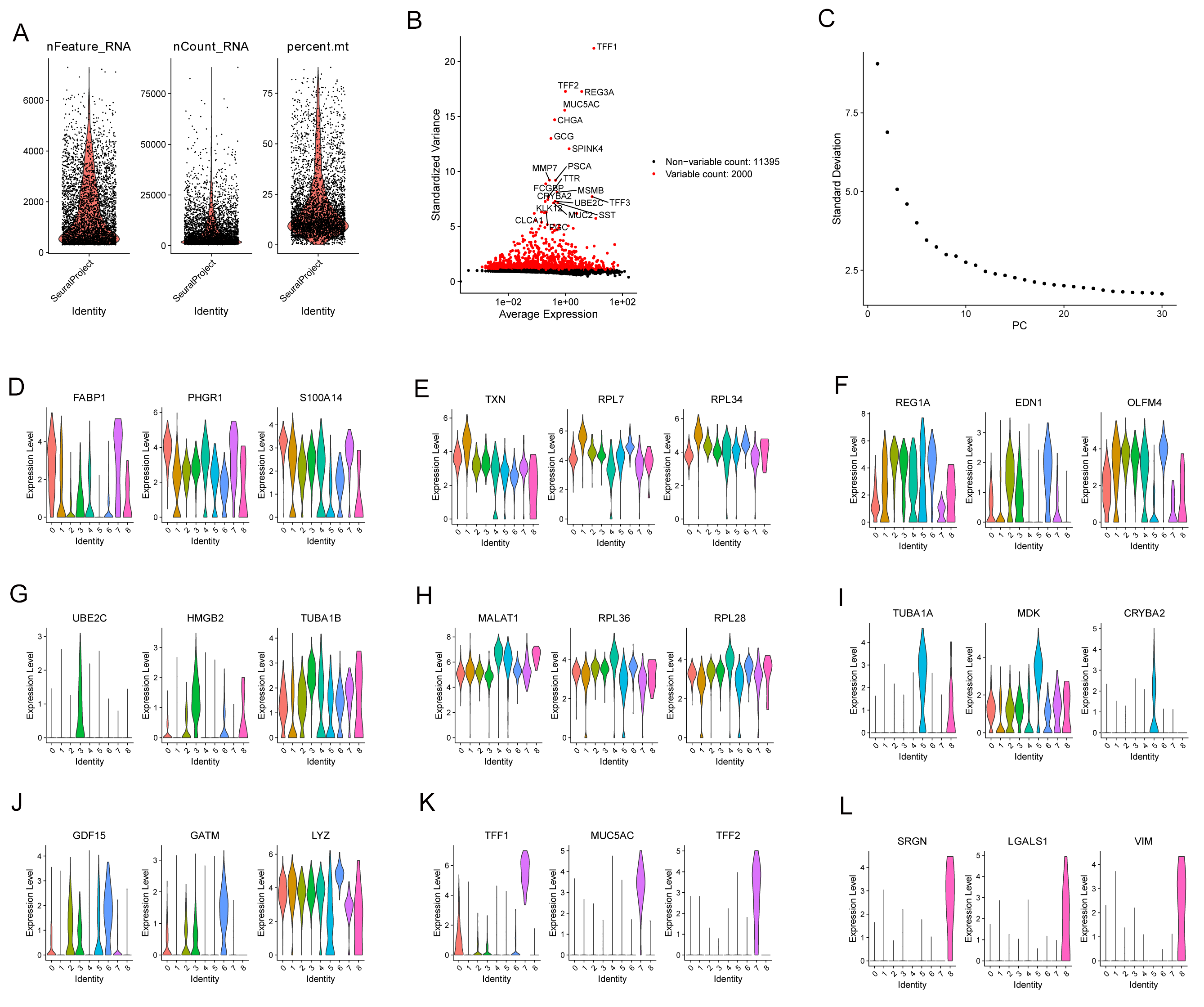

Supplement: Supplementary Figure 1 — (A) Quality control in the single-cell RNA sequencing analysis. (B) The HVGs and the top 20 HVGs were labeled. (C) The standard deviation explained by PC. (D) The top (ranked by log fold change) marker genes for each malignant cell clusters. [file Image_1.TIF]

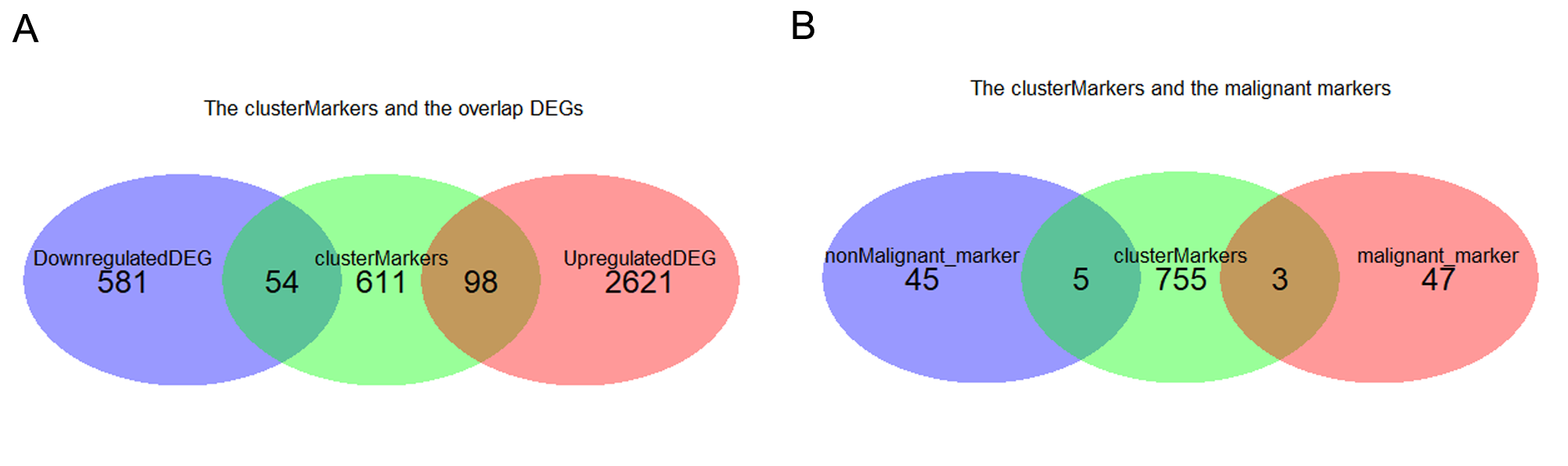

Supplement: Supplementary Figure 2 — (A) Most cluster markers were not included among the overlapping up- or down-regulated genes in STAD. (B) Few malignant and non-malignant cell markers were included among the malignant cell cluster markers. [file Image_2.TIF]
